# Automatic integration using asymptotically optimal adaptive Simpson quadrature

**DOI:** 10.1007/s00211-014-0684-3

**Published:** 2014-11-25

**Authors:** Leszek Plaskota

**Affiliations:** Faculty of Mathematics, Informatics, and Mechanics, University of Warsaw, Banacha 2, 02-097 Warsaw, Poland

**Keywords:** 65Y20, 65D05, 41A10, 41A25

## Abstract

We present a novel theoretical approach to the analysis of adaptive quadratures and adaptive Simpson quadratures in particular which leads to the construction of a new algorithm for automatic integration. For a given function $f\in C^4$ with $f^{(4)}\ge 0$ and possible endpoint singularities the algorithm produces an approximation to $\int _a^bf(x)\,{\mathrm d}x$ within a given $\varepsilon$ asymptotically as $\varepsilon \rightarrow 0$. Moreover, it is optimal among all adaptive Simpson quadratures, i.e., needs the minimal number $n(f,\varepsilon )$ of function evaluations to obtain an $\varepsilon$-approximation and runs in time proportional to $n(f,\varepsilon )$.

## Introduction

Consider a numerical approximation of the integral

1 $$\begin{aligned} I(f)=\int _a^b f(x)\,{\mathrm d}x \end{aligned}$$

for a function $f:[a,b]\rightarrow {\mathbb R}$. Ideally we would like to have an automatic routine that for given $f$ and error tolerance $\varepsilon$ produces an approximation $Q(f)$ to $I(f)$ such that it uses as few function evaluations as possible and its error

$$\begin{aligned} |I(f)-Q(f)|\le \varepsilon . \end{aligned}$$

This is usually realized with the help of *adaption*. Recall a general principle. For a given interval two simple quadrature rules are applied, one more accurate than the other. If the difference between them is sufficiently small, the integral in this interval is approximated by the more accurate quadrature. Otherwise the interval is divided into smaller subintervals and the above rule is recursively applied for each of the subintervals. The oldest and probably most known examples of automatic integration are adaptive Simpson quadratures [8–11], see also [4] for an account on adaptive numerical integration.

An unquestionable advantage of adaptive quadratures is that they try to maintain the error on a prescribed level $\varepsilon$ and simultaneously adjust the length of the successive subintervals to the underlying function. This often results in a much more efficient final subdivision of $[a,b]$ than the nonadaptive uniform subdivision. For those reasons adaptive quadratures are now frequently used in computational practice, and those using higher order *Gauss-Kronrod* rules [1, 5, 15] are standard components of numerical packages and libraries such as MATLAB, NAG or QUADPACK [13]. Nevertheless, to the author’s knowledge, there is no satisfactory and rigorous analysis that would explain good behavior of adaptive quadratures in a quantitative way or identify classes of functions for which they are superior to nonadaptive quadratures. This paper is an attempt to partially fill in this gap.

At this point we have to admit that there are theoretical results showing that adaptive quadratures are not better than nonadaptive quadratures. This holds in the *worst case setting* over convex and symmetric classes of functions. There are also corresponding *adaption-does-not-help* results in other settings, see, e.g., [12, 14, 17, 18]. On the other hand, if the class is not convex and/or a different from the worst case error criterion is used to compare algorithms then adaption can significantly help, see [2] or [16].

In this paper we present a novel theoretical approach to the analysis of adaptive Simpson quadratures. We want to stress that the restriction to the Simpson rule as a basic component of composite rules is only for simplicity and we could equally well use higher order quadratures. The Simpson rule is a relatively simple quadrature and therefore better enables clear development of our ideas. To be more specific, we analyze the adaptive Simpson quadratures from the point of view of computational complexity. Allowing *all* possible subdivision strategies our goal is to find an *optimal* strategy for which the corresponding algorithm returns an $\varepsilon$-approximation to the integral (1) using the minimal number of integrand evaluations or, equivalently, the minimal number of subintervals. The main analysis is *asymptotic* and done assuming that $f$ is four times continuously differentiable and its $4$th derivative is positive.

To reach our goal we first derive formulas for the asymptotic error of adaptive Simpson quadratures. Following [7] we find that the optimal subdivision strategy produces the partition $a=x_0^*<\cdots <x_m^*=b$ such that

$$\begin{aligned} \int _a^{x_i^*}\left( f^{(4)}(x)\right) ^{1/5}\,{\mathrm d}x\,=\, \frac{i}{m}\int _a^b\left( f^{(4)}(x)\right) ^{1/5}\,{\mathrm d}x,\qquad i=0,1,\ldots ,m. \end{aligned}$$

This partition is practically realized by maintaining the error on successive subintervals on the same level. The optimal error corresponding to the subdivision into $m$ subintervals is then proportional to $L^{\mathrm{opt}}(f)\,m^{-4}$ where

$$\begin{aligned} L^{\mathrm{opt}}(f)=\left( \int _a^b\left( f^{(4)}(x)\right) ^{1/5}\,{\mathrm d}x\right) ^5. \end{aligned}$$

For comparison, the errors for the standard adaptive (local) and for nonadaptive (using uniform subdivision) quadratures are respectively proportional to $L^{\mathrm{std}}(f)\,m^{-4}$ and $L^{\mathrm{non}}(f)\,m^{-4}$ where

$$\begin{aligned} L^{\mathrm{std}}(f)\!=(b-a)\left( \int _a^b\left( f^{(4)}(x)\right) ^{1/4}\,{\mathrm d}x\right) ^4\!, \; L^{\mathrm{non}}(f)\!=(b-a)^4\left( \int _a^b\,f^{(4)}(x)\,{\mathrm d}x\!\right) \!. \end{aligned}$$

Obviously, $L^{\mathrm{opt}}(f)\le L^{\mathrm{std}}(f)\le L^{\mathrm{non}}(f)$. Hence the optimal Simpson quadrature is especially effective when $L^{\mathrm{opt}}(f)\ll L^{\mathrm{std}}(f)$. An example is $\int _\delta ^1 x^{-1/2}\,{\mathrm d}x$ with ‘small’ $\delta$. If $\delta =10^{-8}$ then $L^{\mathrm{opt}}(f)$, $L^{\mathrm{std}}(f)$, $L^{\mathrm{non}}(f)$ are correspondingly about $10^5$, $10^8$, $10^{28}$.

Even though the optimal strategy is global it can be efficiently harnessed to automatic integration and implemented in time proportional to $m$. The only serious problem of how to choose the acceptable error $\varepsilon _1$ for subintervals to obtain the final error $\varepsilon$ is resolved by splitting the recursive subdivision process into two phases. In the first phase the process is run with the acceptable error set to a ‘test’ level $\varepsilon _2=\varepsilon$. Then the acceptable error is updated to

$$\begin{aligned} \varepsilon _1=\varepsilon \,m_2^{-5/4} \end{aligned}$$

where $m_2$ is the number of subintervals obtained from the first phase. In the second phase, the recursive subdivision is continued with the ‘target’ error $\varepsilon _1$.

As noted earlier, the main analysis is provided assuming that $f\in C^4([a,b])$ and $f^{(4)}>0$. It turns out that using additional arguments the obtained results can be extended to functions with $f^{(4)}\ge 0$ and/or possible endpoint singularities, i.e., when $f^{(4)}(x)$ goes to $+\infty$ as $x\rightarrow a,b$. For such integrals the optimal strategy works perfectly well while the other quadratures may even lose the convergence rate $m^{-4}$.

The contents of the paper is as follows. In Sect. 2 we recall the standard (local) Simpson quadrature for automatic integration. In Sect. 3 we derive a formula for the asymptotic error of Simpson quadratures and find the optimal subdivision strategy. In Sect. 4 we show how the optimal strategy can be used to construct an optimal algorithm for automatic integration. The final Sect. 5 is devoted to the extensions of the main results. The paper is enriched with numerical tests where the optimal adaptive quadrature is compared with the standard adaptive and nonadaptive quadratures.

We use the following asymptotic notation. For two positive functions of $m$, we write

$$\begin{aligned} \psi _1(m)\lessapprox \psi _2(m) \quad&\text{ iff } \quad \limsup _{m\rightarrow \infty }\frac{\psi _1(m)}{\psi _2(m)}\le 1, \\ \psi _1(m)\approx \psi _2(m) \quad&\text{ iff } \quad \lim _{m\rightarrow \infty }\frac{\psi _1(m)}{\psi _2(m)}=1, \\ \psi _1(m)\preccurlyeq \psi _2(m)\quad&\text{ iff } \quad \limsup _{m\rightarrow \infty }\frac{\psi _1(m)}{\psi _2(m)}<\infty , \\ \psi _1(m)\asymp \psi _2(m) \quad&\text{ iff } \quad 0<\liminf _{m\rightarrow \infty }\frac{\psi _1(m)}{\psi _2(m)}\le \limsup _{m\rightarrow \infty }\frac{\psi _1(m)}{\psi _2(m)}<\infty . \end{aligned}$$

A corresponding notation applies for functions of $\varepsilon$ as $\varepsilon \rightarrow 0$.

## The standard adaptive Simpson quadrature

In its basic formulation the local adaptive Simpson quadrature for automatic integration, which will be called *standard*, can be written recursively as follows. Let Simpson $(u,v,f)$ be the procedure returning the value of the simple three-point Simpson rule on $[u,v]$ for the function $f$, and let $\varepsilon >0$ be the error demand. 
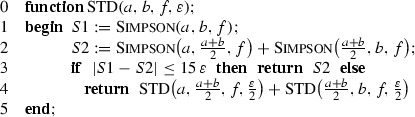
 A justification of STD that can be found in textbooks, e.g., [3, 6], is as follows. Denote by $S_1(u,v;f)$ the three-point Simpson rule,

$$\begin{aligned} S_1(u,v;f)= \left( \frac{u-v}{6}\right) \,\left( f(u)+4\,f\left( \frac{u+v}{2}\right) +f(v)\right) , \end{aligned}$$

and by $S_2(u,v;f)$ the composite Simpson rule that is based on subdivision of $[u,v]$ into two equal subintervals,

$$\begin{aligned} S_2(u,v;f)&= S_1\left( u,\frac{u+v}{2};f\right) +S_1\left( \frac{u+v}{2},v;f\right) \\&= \left( \frac{v-u}{12}\right) \,\left( f(u)+4\,f\left( \frac{3u+v}{4}\right) +2\,f\left( \frac{u+v}{2}\right) \right. \\&\left. +4\,f\left( \frac{u+3v}{4}\right) +f(v)\right) . \end{aligned}$$

We also denote $I(u,v;f)=\int _u^v f(x)\,{\mathrm d}x$. Suppose that

$$\begin{aligned} f\in C^4([a,b]). \end{aligned}$$

If the interval $[u,v]\subseteq [a,b]$ is small enough so that $f^{(4)}$ is ‘almost’ a constant, $f^{(4)}\approx C$ and $C\ne 0$, then

2 $$\begin{aligned} S_2(u,v;f)-S_1(u,v;f)&= (I(u,v;f)-S_1(u,v;f))-(I(u,v;f)-S_2(u,v;f)) \nonumber \\&= \left( -\frac{(v-u)^5}{2880}f^{(4)}(\xi _1)\right) - \left( -\frac{(v-u)^5}{2^4\cdot 2880}f^{(4)}(\xi _2)\right) \nonumber \\&\approx -\frac{(v-u)^5}{2^4\cdot 2880}\,15\,C \nonumber \\&\approx 15\,(I(u,v;f)-S_2(u,v;f)). \end{aligned}$$

Now let $a=x_0<\cdots <x_m=b$ be the final subdivision produced by STD and

$$\begin{aligned} S^{\mathrm{std}}(f;\varepsilon ) \end{aligned}$$

be the result returned by STD. Then, provided the estimate (2) holds for any $[x_{i-1},x_i]$, we have

$$\begin{aligned} |I(f)-S^{\mathrm{std}}(f;\varepsilon )|&\le \sum _{i=1}^m\Big |I(x_{i-1},x_i;f)-S_2(x_{i-1},x_i;f)\Big | \\&\approx \frac{1}{15}\, \sum _{i=1}^m|S_1(x_{i-1},x_i;f)-S_2(x_{i-1},x_i;f)| \\&\le \frac{1}{15}\,\sum _{i=1}^m 15\,\varepsilon \, \frac{x_i-x_{i-1}}{b-a}\,=\,\varepsilon . \end{aligned}$$

This reasoning has a serious defect; namely, the approximate equality (2) can be applied only when the interval $[u,v]$ is sufficiently small. Hence STD can terminate too early and return a completely false result. In an extreme case of $[a,b]=[0,4]$ and $f(x)=\prod _{i=0}^4 (x-i)^2$ we have $I(f)>0$ but STD returns zero independently of how small $\varepsilon$ is. Of course, concrete implementations of STD can be equipped with additional mechanisms to avoid or at least to reduce the probability of such unwanted occurrences. To radically cut the possibility of premature terminations we assume, in addition to $f\in C^4([a,b])$, that the fourth derivative is of constant sign, say,

3 $$\begin{aligned} f^{(4)}(x)>0\qquad \text{ for } \text{ all }\quad x\in [a,b]. \end{aligned}$$

Equivalently, this obviously means that $f^{(4)}(x)\ge c$ for some $c>0$ that depends on $f$. Assumption (3) assures that the maximum length of the subintervals produced by STD decreases to zero as $\varepsilon \rightarrow 0$ and the asymptotic equality (2) holds. Indeed, denote by $D(u,v;f)$ the divided difference of $f$ corresponding to $5$ equispaced points $z_j=u+jh/4$, $0\le j\le 4$, where $h=v-u$, i.e.,

$$\begin{aligned} D(u,v;f)&= f[z_0,z_1,z_2,z_3,z_4] \\&= \frac{32}{3h^4}\left( f(z_0)-4f(z_1)+6f(z_2)-4f(z_3)+f(z_4)\right) . \end{aligned}$$

Since

$$\begin{aligned} S_2(u,v;f)-S_1(u,v;f) = -\frac{h^5}{2^7}\,D(u,v;f), \end{aligned}$$

the termination criterion

$$\begin{aligned} |S_2(u,v;f)-S_1(u,v;f)|\,\le \, 15\,\varepsilon \left( \frac{v-u}{b-a}\right) \end{aligned}$$

that is checked in line 3 of STD for the current subinterval $[u,v]$, is equivalent to

4 $$\begin{aligned} (v-u)^4\,|D(u,v;f)|\,\le \,\frac{15\cdot 2^7}{b-a}\,\varepsilon . \end{aligned}$$

Our conclusion about applicability of (2) follows from the inequality $D(u,v;f)\ge c/4!$

Observe that each splitting of a subinterval $[u,v]$ results in the (asymptotic) decrease of the controlled value in (4) by the factor of $2^4$. Thus the algorithm asymptotically returns the approximation of the integral within $\varepsilon$, as desired. Specifically, we have

5 $$\begin{aligned} \frac{\varepsilon }{16} \;\lessapprox \; S^{\mathrm{std}}(f;\varepsilon )-I(f) \lessapprox \varepsilon \qquad \text{ as }\quad \varepsilon \rightarrow 0. \end{aligned}$$

### *Remark 1*

The inequality (5) explains why numerical tests often show better performance of STD than expected. To avoid this it is suggested to run STD with larger input parameter, say $2\varepsilon$ instead of $\varepsilon$.

## Optimizing the process of interval subdivision

The error formula (5) for the standard adaptive Simpson quadrature does not say anything about how the number $m$ of subintervals depends on $\varepsilon$, or what is the actual error after producing $m$ subintervals. We now study this question for different subdivision strategies. In order to be consistent with STD we assume that for a given subdivision $a=x_0<x_1<\cdots <x_m=b$ we apply $S_2(x_{i-1},x_i;f)$ for each of the subintervals $[x_{i-1},x_i]$, so that the final approximation

$$\begin{aligned} S_m(f)=\sum _{i=1}^m S_2(x_{i-1},x_i;f) \end{aligned}$$

uses $n=4m+1$ function evaluations.

The goal is to find *optimal* strategy, i.e., the one that for any function $f\in C^4([a,b])$ satisfying (3) produces a subdivision for which the error of the corresponding Simpson quadrature $S_m(f)$ is asymptotically minimal (as $m\rightarrow \infty$).

We first analyze two particular strategies, nonadaptive and standard adaptive, and then derive the optimal strategy. In what follows, the constant

$$\begin{aligned} \gamma =\frac{1}{2^4\cdot 2880}=\frac{1}{46\,080}\cong 2.17\times 10^{-5}. \end{aligned}$$

In the nonadaptive strategy, the interval $[a,b]$ is divided into $m$ equal subintervals $[x_{i-1},x_i]$ with $x_i=a+ih$, $h=(b-a)/m$. Let the corresponding Simpson quadrature be denoted by $S_m^{\mathrm{non}}$. Then

6 $$\begin{aligned} S^\mathrm{non}_m(f)-I(f)&= \gamma \,(b-a)^4\, \left( \frac{1}{m}\,\sum _{i=1}^m f^{(4)}(\xi _i)\right) \,m^{-4} \quad \quad (\xi _i\in [x_{i-1},x_i])\nonumber \\&\approx \gamma \,(b-a)^4\, \left( \int _a^b f^{(4)}(x)\,{\mathrm d}x\right) \,m^{-4} \end{aligned}$$

as $$m\rightarrow \infty $$.

Observe that for the asymptotic equality (6) to hold we do not need to assume (3).

We now analyze the standard adaptive strategy used by STD. To do this, we first need to rewrite STD in an equivalent way, where the input parameter is $m$ instead of $\varepsilon$. We have the following *greedy* algorithm.

The algorithm starts with the initial subdivision $a=x_0^{(1)}<x_1^{(1)}=b$. In the $(k+1)$st step, from the current subdivision $a=x_0^{(k)}<\cdots <x_k^{(k)}=b$ a subinterval $[x_{i^*-1}^{(k)},x_{i^*}^{(k)}]$ is selected with the highest value

7 $$\begin{aligned} \left( x_i^{(k)}-x_{i-1}^{(k)}\right) ^4\, \left| D\left( x_{i-1}^{(k)},x_i^{(k)};f\right) \right| ,\qquad 1\le i\le k, \end{aligned}$$

and the midpoint $(x_{i^*-1}^{(k)}+x_{i^*}^{(k)})/2$ is added to the subdivision.

Denote by $S_m^{\mathrm{std}}(f)$ the result returned by the corresponding Simpson quadrature when applied to $m$ subintervals. Then, in view of (4), the values $S_m^{\mathrm{std}}(f)$ and $S^{\mathrm{std}}(f;\varepsilon )$ are related as follows. Let $m=m(\varepsilon )$ be the minimal number of steps after which (4) is satisfied by each of the subintervals $[x^{(m)}_{i-1},x^{(m)}_i]$. Then

8 $$\begin{aligned} S_m^{\mathrm{std}}(f)=S^{\mathrm{std}}(f;\varepsilon ). \end{aligned}$$

We are ready to show the error formula for $S_m^{\mathrm{std}}$ corresponding to (6).

### **Theorem 1**

Let $f\in C^4([a,b])$ and $f^{(4)}(x)>0$ for all $x\in [a,b]$. Then

$$\begin{aligned} S_m^\mathrm{std}(f)-I(f) \,\asymp \, \gamma \,(b-a) \left( \int _a^b \left( f^{(4)}(x)\right) ^{1/4}\,{\mathrm d}x\right) ^4\,m^{-4} \qquad \text{ as }\quad m\rightarrow \infty . \end{aligned}$$

### *Proof*

We fix $\ell$ and divide the interval $[a,b]$ into $2^\ell$ equal subintervals $[z_{i-1},z_i]$ of length $(b-a)/2^\ell$. Call this partition a coarse grid, in contrast to the fine grid produced by $S_m^\mathrm{std}$. Let

$$\begin{aligned} C_i=\max _{z_{i-1}\le x\le z_i} f^{(4)}(x),\qquad c_i=\min _{z_{i-1}\le x\le z_i} f^{(4)}(x). \end{aligned}$$

Let $m$ be sufficiently large, so that the fine grid contains all the points of the coarse grid. Denote by $z_{i-1}=x_{i,0}<x_{i,1}<\cdots <x_{i,m_i}=z_i$ the points of the fine grid contained in the $i$th interval of the coarse grid, and $h_{i,j}=x_{i,j}-x_{i,j-1}$. Then the error can be bounded from below as

$$\begin{aligned} S_m^{\mathrm{std}}(f)-I(f)&= \sum _{i=1}^{2^\ell }\sum _{j=1}^{m_i} \left( S_2(x_{i,j-1},x_{i,j};f)-I(x_{i,j-1};x_{i,j};f)\right) \\&= \gamma \,\sum _{i=1}^{2^\ell }\sum _{j=1}^{m_i} h_{i,j}^5 f^{(4)}(\xi _{i,j}) \quad \quad (\xi _{i,j}\in [x_{i,j-1},x_{i,j}])\\&\ge \gamma \,\sum _{i=1}^{2^\ell }\sum _{j=1}^{m_i} h_{i,j}^5c_i. \end{aligned}$$

Suppose for a moment that for all $i,j$ we have $h_{i,j}^4c_i=A$ for some $A$. Then $(b-a)/2^\ell =\sum _{j=1}^{m_i}h_{i,j}=m_i(A/c_i)^{1/4}$. Using $\sum _{i=1}^{2^\ell }m_i=m$ we get

$$\begin{aligned} A=\left( \frac{b-a}{2^\ell }\right) ^4 \left( \sum _{i=1}^{2^\ell } c_i^{1/4}\right) ^4\,m^{-4}. \end{aligned}$$

Observe now that any splitting of a subinterval decreases $h_{i,j}^4c_i$ by the factor of $16$. Hence

$$\begin{aligned} \frac{\max _{i,j}h_{i,j}^4c_i}{\min _{i,j}h_{i,j}^4c_i}\le 16 \end{aligned}$$

and consequently $h_{i,j}^4c_i\ge A/16$ for all $i,j$. Thus

$$\begin{aligned} S_m^{\mathrm{std}}(f)-I(f)&\ge \gamma \,\sum _{i=1}^{2^\ell } \sum _{j=1}^{m_i}h_{i,j}\frac{A}{16} \,=\, \frac{1}{16}\gamma \,(b-a)\,A \\&= \frac{1}{16}\gamma \,(b-a)\left( \frac{b-a}{2^\ell }\right) ^4 \left( \sum _{i=1}^{2^\ell }c_i^{1/4}\right) ^4\,m^{-4}. \end{aligned}$$

To obtain the upper bound, we proceed similarly. Replacing $c_i$ with $C_i$ and using the equation $h_{i,j}^4C_i\le 16 A$ we get that

$$\begin{aligned} S_m^{\mathrm{std}}(f)-I(f) \,\le \, 16\gamma \,(b-a)\left( \frac{b-a}{2^\ell }\right) ^4 \left( \sum _{i=1}^{2^\ell }C_i^{1/4}\right) ^4\,m^{-4}. \end{aligned}$$

To complete the proof we notice that both

$$\begin{aligned} \sum _{i=1}^{2^\ell }c_i^{1/4}(b-a)2^{-\ell } \quad \text{ and }\quad \sum _{i=1}^{2^\ell }C_i^{1/4}(b-a)2^{-\ell } \end{aligned}$$

are Riemann sums that converge to the integral $\int _a^b\left( f^{(4)}(x)\right) ^{1/4}\!\,{\mathrm d}x$ as $\ell \rightarrow \infty$. $\square$

### *Remark 2*

From the proof it follows that the constants in the ‘$\asymp$’ notation in Theorem 1 are asymptotically between $1/16$ and $16$. The gap between the upper and lower constants is certainly much overestimated, see also Remark 4.

The two strategies, nonadaptive and standard adaptive, will be used as reference points for comparison with the optimal strategy that we now derive. We first allow *all* possible subdivisions of $[a,b]$ regardless of the possibility of their practical realization.

### **Proposition 1**

The subdivision determined by points

$$\begin{aligned} a=x_0^*<x_2^*<\cdots <x_m^*=b \end{aligned}$$

where $$x_i^*$$ satisfy

$$\begin{aligned} \int _a^{x_i^*}\left( f^{(4)}(x)\right) ^{1/5}\,{\mathrm d}x=\frac{i}{m}\, \int _a^b\left( f^{(4)}(x)\right) ^{1/5}\,{\mathrm d}x,\qquad i=0,1,2,\ldots ,m, \end{aligned}$$

is optimal. For the corresponding quadrature $S_m^*$ we have

$$\begin{aligned} S_m^*(f)-I(f)\approx \gamma \, \left( \int _a^b \left( f^{(4)}(x)\right) ^{1/5}\,{\mathrm d}x\right) ^5\,m^{-4} \qquad \text{ as }\quad m\rightarrow \infty . \end{aligned}$$

### *Proof*

We first show the lower bound. Let $S_m$ be the Simpson quadrature that is based on an arbitrary subdivision. Proceeding as in the beginning of the proof of Theorem 1 we get that for sufficiently large $m$ the error of $S_m$ is lower bounded by

$$\begin{aligned} S_m(f)-I(f)\,\ge \,\gamma \,\sum _{i=1}^{2^\ell } \sum _{j=1}^{m_i} h_{i,j}^5c_i\,\ge \,\gamma \, \left( \frac{b-a}{2^\ell }\right) ^5\sum _{i=1}^{2^\ell }\frac{c_i}{m_i^4}, \end{aligned}$$

where $m_i$ is the number of subintervals of the fine grid in the *i*th subinterval of the coarse grid. (We assume without loss of generality that the coarse grid is contained in the fine grid.) Minimizing this with respect to $m_i$ such that $\sum _{i=1}^{2^\ell }m_i=m$ we obtain the optimal values

$$\begin{aligned} m_i^*=\left( \frac{c_i^{1/5}}{\sum _{i=1}^{2^\ell }c_i^{1/5}}\right) \,m, \qquad 1\le i\le 2^\ell . \end{aligned}$$

After substituting $m_i$ with $m_i^*$ in the error formula we finally get

9 $$\begin{aligned} \nonumber S_m(f)-I(f)&\ge \gamma \,\left( \frac{b-a}{2^\ell }\right) ^5 \left( \sum _{i=1}^{2^\ell }c_i^{1/5}\right) ^5\,m^{-4} \\&\approx \gamma \, \left( \int _a^b\left( f^{(4)}(x)\right) ^{1/5}\,{\mathrm d}x\right) ^5\,m^{-4}. \end{aligned}$$

Since for the optimal $m_i^*$ we have

10 $$\begin{aligned} h^*_{i,j}c_i^{1/5}=\left( \frac{b-a}{2^\ell m}\right) \left( \sum _{i=1}^{2^\ell }c_i^{1/5}\right) , \end{aligned}$$

the lower bound (9) is attained by the subdivision determined by $\{x_i^*\}$. $\square$

Now the question is whether the optimal subdivision into $m$ subintervals of Proposition 1 can be practically realized, i.e., using $4m+1$ function evaluations. The answer is positive, at least up to an absolute constant. The corresponding algorithm $S_m^\mathrm{opt}$ runs as $S_m^\mathrm{std}$ with the only difference that in each step it halves the subinterval with the highest value

11 $$\begin{aligned} \left( x_i^{(k)}-x_{i-1}^{(k)}\right) ^5\, \left| D\left( x_{i-1}^{(k)},x_i^{(k)};f\right) \right| ,\qquad 1\le i\le k \end{aligned}$$

[instead of (7)].

### **Theorem 2**

Let $f\in C^4([a,b])$ and $f^{(4)}(x)>0$ for all $x\in [a,b]$. Then

$$\begin{aligned} S_m^\mathrm{opt}(f)-I(f) \,\lessapprox \,K\,\gamma \, \left( \int _a^b \left( f^{(4)}(x)\right) ^{1/5}\,{\mathrm d}x\right) ^5\,m^{-4} \qquad \text{ as }\quad m\rightarrow \infty \end{aligned}$$

where $$K\le 32$$.

### *Proof*

The proof goes as the proof of the upper bound of Theorem 1 with obvious changes related to the facts that now the algorithm tries to balance (11) [instead of (7)], and that

$$\begin{aligned} \frac{\max _{i,j} h^5_{i,j}C_i}{\min _{i,j} h_{i,j}^5C_i} \le 32. \end{aligned}$$

$$\square $$

### *Remark 3*

The best constant $K$ of Theorem 2 is certainly much less than 32, see also Remark 4.

We summarize the results of this section. All the three quadratures $S_m^{\mathrm{non}}$, $S_m^{\mathrm{std}}$, $S_m^{\mathrm{opt}}$ converge at rate $m^{-4}$ but the asymptotic constants depend on the integrand $f$ through the multipliers

$$\begin{aligned} L^{\mathrm{non}}(f)&= (b-a)^4\, \left( \int _a^b f^{(4)}(x)\,{\mathrm d}x\right) , \\ L^{\mathrm{std}}(f)&= (b-a)\, \left( \int _a^b\left( f^{(4)}(x)\right) ^{1/4}\,{\mathrm d}x\right) ^4, \\ L^{\mathrm{opt}}(f)&= \left( \int _a^b\left( f^{(4)}(x)\right) ^{1/5}\,{\mathrm d}x\right) ^5. \end{aligned}$$

These multipliers indicate how difficult a function is to integrate using a given quadrature. Obviously, by Hölder’s inequality we have

$$\begin{aligned} L^{\mathrm{opt}}(f)\,\le \,L^{\mathrm{std}}(f)\,\le \,L^{\mathrm{non}}(f). \end{aligned}$$

### *Example 1*

Consider the integral

$$\begin{aligned} I_\delta =\int _\delta ^1 \frac{1}{2\sqrt{x}}\,{\mathrm d}x \qquad \text{ with }\quad 0<\delta <1. \end{aligned}$$

In this case $L^{\mathrm{non}}$, $L^{\mathrm{std}}$, $L^{\mathrm{opt}}$ rapidly increase as $\delta$ decreases, as shown in Table 1.

**Table 1:** Values of $L^{\mathrm{non}}$, $L^{\mathrm{std}}$, $L^{\mathrm{opt}}$ for $\int _\delta ^1\tfrac{1}{2\sqrt{x}}\,{\mathrm d}x$ with different $\delta$

$\delta$	$L^{\mathrm{non}}$	$L^{\mathrm{std}}$	$L^{\mathrm{opt}}$
$0.5$	$6.04\times {10}^{-1}$	$4.51\times {10}^{-1}$	$4.41\times {10}^{-1}$
$10^{-2}$	$9.01\times {10}^{06}$	$4.88\times {10}^{03}$	$2.24\times {10}^{03}$
$10^{-4}$	$9.37\times {10}^{13}$	$2.94\times {10}^{05}$	$2.59\times {10}^{04}$
$10^{-8}$	$9.38\times {10}^{27}$	$8.82\times {10}^{07}$	$1.39\times {10}^{05}$
$10^{-16}$	$9.38\times {10}^{55}$	$1.29\times {10}^{12}$	$2.89\times {10}^{05}$

Numerical computations confirm the theory very well. We tested all the three quadratures (the adaptive quadratures being implemented in $m\log m$ running time using *heap* data structure) and ran them for different values of $\delta$. Specific results are as follows.

For $\delta =0.5$ the quadratures $S_m^{\mathrm{non}}$, $S_m^{\mathrm{std}}$, and $S_m^{\mathrm{opt}}$ give almost identical results independently of $m$. For instance, for $m=10^2$ the errors are respectively $1.31\times 10^{-13}$, $1.46\times 10^{-13}$, $1.46\times 10^{-13}$, and for $m=10^3$ we have $1.28\times 10^{-17}$, $1.43\times 10^{-17}$, $1.35\times 10^{-17}$. Note that the smallest error for the nonadaptive quadrature is caused by the fact that $S_m^{\mathrm{non}}$ has a little better absolute constant in the error formula (6) than the adaptive quadratures.

**Fig. 1:**
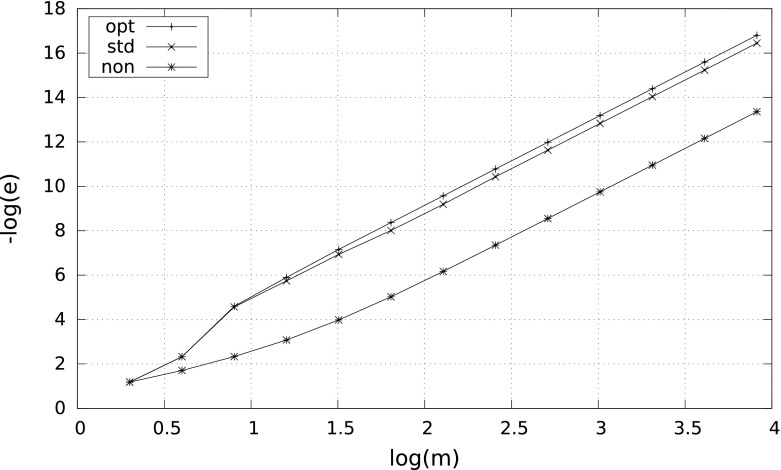
Error $e$ versus $m$ for $\int _\delta ^1\tfrac{1}{2\sqrt{x}}\,{\mathrm d}x$ with $\delta =10^{-2}$

**Fig. 2:**
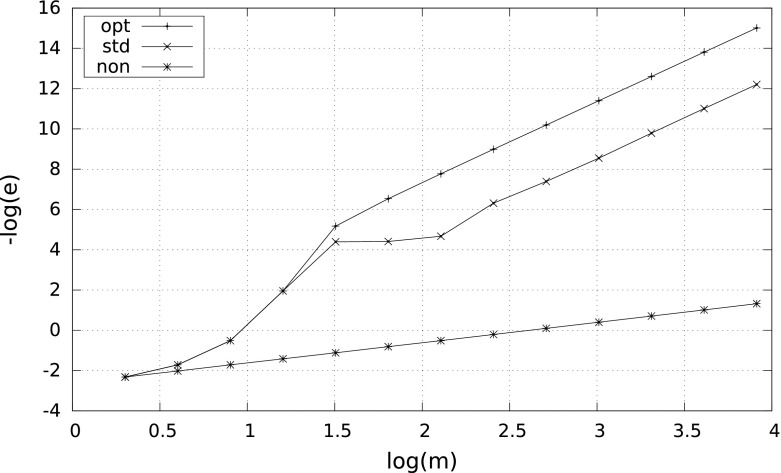
Error $e$ versus $m$ for $\int _\delta ^1\tfrac{1}{2\sqrt{x}}\,{\mathrm d}x$ with $\delta =10^{-8}$

However, the smaller $\delta$, the more differences between the results. A characteristic behavior of the errors for $\delta =10^{-2}$ and $\delta =10^{-8}$ is illustrated by Figs. 1 and 2. Observe that in case $\delta =10^{-8}$ the nonadaptive quadrature needs more than $10^4$ subintervals to reach the right convergence rate $m^{-4}$.

### *Remark 4*

It is interesting to see the behavior of

$$\begin{aligned} K_m^\mathrm{qad}(f)\,=\, \frac{\left( S_m^\mathrm{qad}(f)-I(f)\right) \cdot m^4}{\gamma \cdot L^\mathrm{qad}(f)}, \qquad \mathrm{qad}\in \{\mathrm{non},\mathrm{std},\mathrm{opt}\}. \end{aligned}$$

**Fig. 3:**
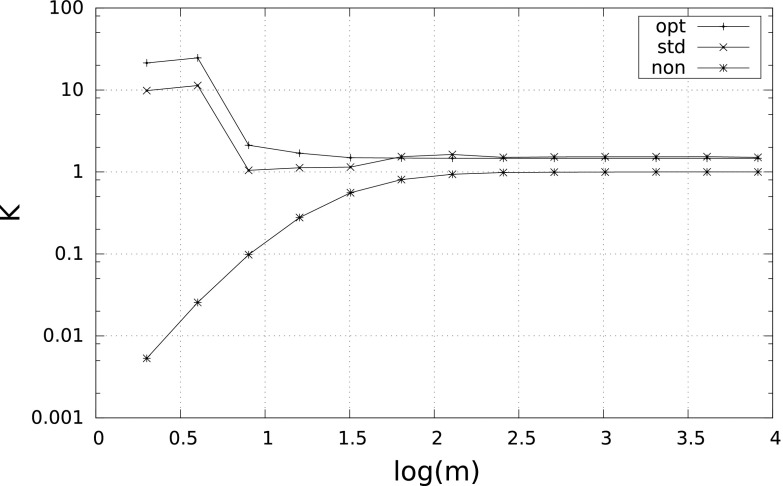
$K_m^{\mathrm{non}}$, $K_m^{\mathrm{std}}$, $K_m^{\mathrm{opt}}$ versus $m$ for $\int _\delta ^1\tfrac{1}{2\sqrt{x}}\,{\mathrm d}x$ with $\delta =10^{-2}$

By (6) we have that $\lim _{m\rightarrow \infty }K_m^{\mathrm{non}}(f)=1$. The corresponding limits for the adaptive quadratures are unknown; however, we ran some numerical tests and we never obtained more than $1.5$. This would mean, in particular, that $S_m^{\mathrm{opt}}$ is at most $50~\%$ worse than $S_m^*$. Figure 3 shows the behavior of $K_m^\mathrm{qad}(f)$ for the integral $I_\delta$ of Example 1 with $\delta =10^{-2}$.

## Automatic integration using optimal subdivision strategy

We want to have an algorithm that automatically computes an integral within a given error tolerance $\varepsilon$. An example of such algorithm is the recursive STD. Recall that the recursive nature of STD allows to implement it in time proportional to the number $m$ of subintervals using *stack* data structure. However, it does not use the optimal subdivision strategy. On the other hand, the algorithm $S_m^{\mathrm{opt}}$ uses the optimal strategy, but one does not know in advance how large $m$ should be to have the error $|S_m^{\mathrm{opt}}(f)-I(f)|\le \varepsilon$. In addition, the best implementation of $S_m^{\mathrm{opt}}$ (that uses heap data structure) runs in time proportional to $m\log m$. Thus the question now is whether there exists an algorithm that runs in time linear in $m$ and produces an approximation to the integral within $\varepsilon$ using the optimal subdivision strategy.

Since the optimal subdivision is such that the errors on subintervals are roughly equal, the suggestion is that one should run STD with the only difference that it is recursively called with parameter $\varepsilon$ instead of $\varepsilon /2$. Denote such modification by OPT. 
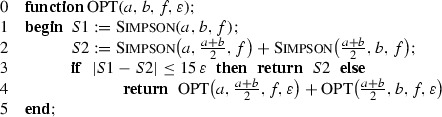
 Let

$$\begin{aligned} S^{\mathrm{opt}}(f;\varepsilon ) \end{aligned}$$

be the result returned by OPT. Analogously to (8) we have

$$\begin{aligned} S_m^{\mathrm{opt}}(f)=S^{\mathrm{opt}}(f;\varepsilon ) \end{aligned}$$

if $m$ is the minimal number of steps after which (11) is satisfied by all subintervals.

It is clear that OPT does not return an $\varepsilon$-approximation when $\varepsilon$ is the input parameter. However we are able to estimate *a posteriori* error. Indeed, let $m_1$ be the number of subintervals produced by OPT for an $\varepsilon _1$. Then

12 $$\begin{aligned} \frac{m_1\,\varepsilon _1}{32}\,\lessapprox \, S^\mathrm{opt}(f;\varepsilon _1)-I(f) \,\lessapprox \, m_1\,\varepsilon _1\qquad \text{ as }\quad \varepsilon _1\rightarrow 0. \end{aligned}$$

We need to find $\varepsilon _1$ such that $m_1\varepsilon _1\le \varepsilon$. Since $m_1$ depends not only on $\varepsilon _1$ but also on $L^{\mathrm{opt}}(f)$, it seems hopeless to predict $\varepsilon _1$ in advance. Surprisingly this is not true.

The idea of the algorithm is as follows. We first run OPT with some $\varepsilon _2\le \varepsilon$ obtaining a subdivision consisting of $m_2$ subintervals. Next, using (12) and Theorem 2 we estimate $L^{\mathrm{opt}}(f)$, and using again Theorem 2 we find the ‘right’ $\varepsilon _1$. Finally OPT is resumed with the input $\varepsilon _1$ and with subdivision obtained in the preliminary run of OPT. As we shall see later, this idea can be implemented in time proportional to $m_1$.

Concrete calculations are as follows. From the equality

$$\begin{aligned} \alpha _2\,m_2\,\varepsilon _2\,=\, S^{\mathrm{opt}}_{m_2}(f)-I(f)\,=\, K_2\,\gamma \, L^{\mathrm{opt}}(f)\, m_2^{-4} \end{aligned}$$

where $\alpha _2$ and $K_2$ depend on $\varepsilon _2$, we have

13 $$\begin{aligned} L^{\mathrm{opt}}(f)\,=\,\frac{\alpha _2}{K_2\gamma }\,\varepsilon _2\, m_2^5. \end{aligned}$$

We need $\varepsilon _1$ such that for the corresponding $m_1$ the error of $S^{\mathrm{opt}}_{m_1}(f)$ is at most $\varepsilon$, i.e.,

$$\begin{aligned} \alpha _1\,m_1\varepsilon _1\,=\, S^{\mathrm{opt}}_{m_1}(f)-I(f)\,=\,K_1\,\gamma \, L^{\mathrm{opt}}(f)\, m_1^{-4} \,\le \,\varepsilon \end{aligned}$$

where $\alpha _1$ and $K_1$ depend on $\varepsilon _1$. Substituting $L^{\mathrm{opt}}(f)$ with the right hand side of (13) we obtain

14 $$\begin{aligned} m_1=m_2\left( \frac{K_1\,\alpha _2\,\varepsilon _2}{K_2\,\alpha _1\,\varepsilon _1}\right) ^{1/5}, \end{aligned}$$

and solving the inequality $\alpha _1m_1\varepsilon _1\le \varepsilon$ with $m_1$ given by (14) we get

$$\begin{aligned} \varepsilon _1\,\le \,\beta \left( \frac{\varepsilon ^5}{\varepsilon _2\, m_2^5}\right) ^{1/4}\qquad \text{ where }\quad \beta =\left( \frac{K_2}{K_1\,\alpha _1^4\,\alpha _2}\right) ^{1/4}. \end{aligned}$$

Recall that, asymptotically, $\alpha _1$ and $\alpha _2$ are in $[1/32,1]$ which means that $\beta$ can be asymptotically bounded from below by $1$. Hence, taking

15 $$\begin{aligned} \varepsilon _2=\varepsilon \qquad \text{ and }\qquad \varepsilon _1=\varepsilon \, m_2^{-5/4} \end{aligned}$$

we have

$$\begin{aligned} S^{\mathrm{opt}}(f;\varepsilon _1)-I(f)\,\lessapprox \,\varepsilon \qquad \text{ as }\quad \varepsilon \rightarrow 0. \end{aligned}$$

The choice of $\varepsilon _1$ given by (15) is rather conservative. In practice, we observe that the error of $S^{\mathrm{opt}}(f;\varepsilon _1)$ is ‘on average’ even $6$ or more times smaller than $\varepsilon$. Hence we encounter the same phenomenon as for the standard Simpson quadrature, see Remark 1. Yet, in the latter case, the error is usually not so much smaller than $\varepsilon$. As a consequence, for integrands $f$ with $L^{\mathrm{opt}}(f)\cong L^{\mathrm{std}}(f)$ the approximation $S^{\mathrm{std}}(f;\varepsilon )$ may use less subintervals than $S^{\mathrm{opt}}(f;\varepsilon _1)$.

To avoid an excessive work, we propose to run the optimal algorithm with the input $B\,\varepsilon _1$ instead of $\varepsilon _1$ where, say,

$$\begin{aligned} B=4^{5/4}=4\,\sqrt{2}\,\cong \, 5.656854 \end{aligned}$$

(This corresponds to $\alpha _1,\alpha _2=1/4$.) We stress that such choice of $B$ is based on some heuristics and is not justified by any rigorous arguments.

### *Example 2*

We present test results for the integral $I_\delta =\int _\delta ^1 x^{-1/2}/2\,{\mathrm d}x$ of Example 1 with $\delta =10^{-2}$ and $\delta =10^{-8}$, for the standard and optimal Simpson quadratures. In Tables 2 and 3 the results are given correspondingly for $S^{\mathrm{std}}(f;\varepsilon )$ and $S^{\mathrm{opt}}(f;\varepsilon _1)$, while in Tables 4 and 5 for $S^{\mathrm{std}}(f;2\varepsilon )$ and $S^{\mathrm{opt}}(f;4\sqrt{2}\varepsilon _1)$.

**Table 2:** Standard and optimal quadratures for $\int _\delta ^1\tfrac{1}{2\sqrt{x}}\,{\mathrm d}x$ with $\delta =10^{-2}$

$\varepsilon$	$\text{ Standard }\;(\mathrm{B}=1)$		$\text{ Optimal }\;(\mathrm{B}=1)$	
	Error	$m$	Error	$m$
1.0E$-$03	3.54064E$-$05	13	1.28793E$-$04	11
1.0E$-$04	2.70762E$-$05	15	1.01889E$-$05	19
1.0E$-$05	1.88171E$-$06	29	1.63224E$-$06	29
1.0E$-$06	4.21492E$-$07	47	1.36983E$-$07	53
1.0E$-$07	3.76521E$-$08	89	1.04855E$-$08	101
1.0E$-$08	3.02315E$-$09	165	1.13726E$-$09	177
1.0E$-$09	3.10104E$-$10	295	1.12420E$-$10	317
1.0E$-$10	3.44621E$-$11	523	1.20113E$-$11	555
1.0E$-$11	3.62842E$-$12	923	1.19899E$-$12	987
1.0E$-$12	3.56781E$-$13	1,627	1.19112E$-$13	1,757

**Table 3:** Standard and optimal quadratures for $\int _\delta ^1\tfrac{1}{2\sqrt{x}}\,{\mathrm d}x$ with $\delta =10^{-8}$

$\varepsilon$	$\text{ Standard }\;(\mathrm{B}=1)$		$\text{ Optimal }\;(\mathrm{B}=1)$	
	Error	$m$	Error	$m$
1.0E$-$03	3.95465E$-$05	107	1.01415E$-$04	43
1.0E$-$04	3.34721E$-$05	189	1.02928E$-$05	61
1.0E$-$05	2.32107E$-$06	341	1.02185E$-$06	95
1.0E$-$06	3.69227E$-$07	605	1.25590E$-$07	157
1.0E$-$07	4.06133E$-$08	1,075	1.11498E$-$08	283
1.0E$-$08	3.09464E$-$09	1,905	1.20507E$-$09	491
1.0E$-$09	2.87135E$-$10	3,383	1.15265E$-$10	883
1.0E$-$10	3.48973E$-$11	6,035	1.13688E$-$11	1,577
1.0E$-$11	3.57812E$-$12	10,747	1.16955E$-$12	2,789
1.0E$-$12	3.60253E$-$13	19,123	1.18105E$-$13	4,945

**Table 4:** Standard and optimal quadratures for $\int _\delta ^1\tfrac{1}{2\sqrt{x}}\,{\mathrm d}x$ with $\delta =10^{-2}$

$\varepsilon$	$\text{ Standard }\;(\mathrm{B}=2)$		$\text{ Optimal }\;(\mathrm{B}=4\sqrt{2})$	
	Error	$m$	Error	$m$
1.0E$-$03	1.28793E$-$04	11	8.51947E$-$04	9
1.0E$-$04	2.70762E$-$05	15	3.54064E$-$05	13
1.0E$-$05	1.23725E$-$05	25	+ 6.03436E$-$06	21
1.0E$-$06	4.21492E$-$07	47	7.11390E$-$07	35
1.0E$-$07	5.33769E$-$08	77	6.93759E$-$08	63
1.0E$-$08	5.87002E$-$09	139	6.74705E$-$09	113
1.0E$-$09	6.71603E$-$10	245	6.56445E$-$10	203
1.0E$-$10	6.99015E$-$11	435	6.48708E$-$11	363
1.0E$-$11	6.87621E$-$12	773	6.67488E$-$12	643
1.0E$-$12	6.65216E$-$13	1,383	6.70510E$-$13	1,143

**Table 5:** Standard and optimal quadratures for $\int _\delta ^1\tfrac{1}{2\sqrt{x}}\,{\mathrm d}x$ with $\delta =10^{-8}$

$\varepsilon$	$\text{ Standard }\;(\mathrm{B}=2)$		$\text{ Optimal }\;(\mathrm{B}=4\sqrt{2})$	
	Error	$m$	Error	$m$
1.0E$-$03	3.98407E$-$05	95	1.10755E$-$03	37
1.0E$-$04	3.68038E$-$05	161	1.01415E$-$04	43
1.0E$-$05	1.34783E$-$05	287	5.78319E$-$06	65
1.0E$-$06	4.88650E$-$07	511	7.40987E$-$07	103
1.0E$-$07	5.88129E$-$08	899	6.83728E$-$08	181
1.0E$-$08	6.21797E$-$09	1,603	6.70486E$-$09	321
1.0E$-$09	7.10367E$-$10	2,855	6.71221E$-$10	569
1.0E$-$10	7.42057E$-$11	5,083	6.49621E$-$11	1,019
1.0E$-$11	7.12978E$-$12	9,039	6.59023E$-$12	1,805
1.0E$-$12	6.66354E$-$13	16,031	6.50999E$-$13	3,223

We end this section by presenting a rather detailed description of the optimal algorithm for automatic integration that runs in time proportional to $m_1$. It uses two stacks, $\mathrm {Stack1}$ and $\mathrm {Stack2}$, corresponding to the two phases of the algorithm. The elements of the stacks, $\mathrm {elt}$, $\mathrm {elt1}$, $\mathrm {elt2}$, represent subintervals. Each such element consists of $6$ fields containing information about: the endpoints of the subinterval, function values at the endpoints and at the midpoint, and the value of the three-point Simpson quadrature for this subinterval. Such structure enables evaluation of $f$ only once at each sample point. $\mathrm {Push}$ and $\mathrm {Pop}$ are usual stack commands for inserting and removing elements. 
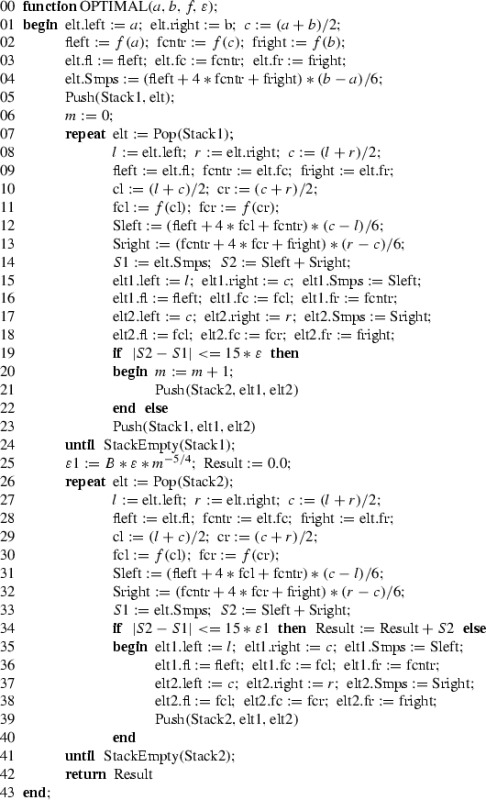


## Extensions: $f^{(4)}\ge 0$ and endpoint singularities

We have analyzed adaptive Simpson quadratures assuming that $f\in C^4([a,b])$ and $f^{(4)}>0$. It turns out that the obtained results hold and automatic integration can be successfully applied also for functions with $f^{(4)}\ge 0$ and functions with endpoint singularities. An observed good behavior of adaptive quadratures for such functions cannot be explained using directly previous tools. What we need is a non-asymptotic error bound for $S_2(u,v;f)$. Such a bound, together with the corresponding result for $S_1(u,v;f)$, is provided by the following lemma.

### **Lemma 1**

Suppose that $f\in C([u,v])$ and $f\in C^4([u_1,v_1])$ for all $u<u_1<v_1<v$. If, in addition, $f^{(4)}(x)\ge 0$ for all $x\in (u,v)$, then

$$\begin{aligned} 1\le \frac{S_1(u,v;f)-I(u,v;f)}{S_1(u,v;f)-S_2(u,v;f)} \le 2 \end{aligned}$$

and

$$\begin{aligned} 0\,\le \,\frac{S_2(u,v;f)-I(u,v;f)}{S_1(u,v;f)-S_2(u,v;f)}\,\le \,1 \end{aligned}$$

(with convention that $0/0=1$).

### *Proof*

Given $c\in (u,v)$, we have that for any $x\in [u,v]$

16 $$\begin{aligned} f(x)=T_c(x)+\int _c^x\frac{(x-t)^3}{3!} f^{(4)}(t)\,{\mathrm d}t, \end{aligned}$$

where $T_c$ is a Taylor polynomial for $f$ of degree $3$ at $c$. (The formula is obvious for $x\in (a,b)$ and by continuity of $f$ it extends to $x=u,v$.) Furthermore, integrating (16) with respect to $x$ we get that

17 $$\begin{aligned} \int _u^v f(x)\,{\mathrm d}x \,=\, \int _u^v T_c(x)\,{\mathrm d}x + \int _u^c\frac{(u-t)^4}{4!} f^{(4)}(t)\,{\mathrm d}t + \int _c^v\frac{(v-t)^4}{4!} f^{(4)}(t)\,{\mathrm d}t. \end{aligned}$$

Using (16) for $z_j=u+jh/4$, $0\le j\le 4$, $h=v-u$, we then obtain

18 $$\begin{aligned} S_1(u,v;f)-S_2(u,v;f)=\frac{h^5}{2^7}D(u,v;f)= \int _u^v \psi _0(u,v;t) f^{(4)}(t)\,{\mathrm d}t \end{aligned}$$

with the Peano kernel $\psi _0(u,v;t)=h^4\Psi _0((t-u)/h)$ where

$$\begin{aligned} \Psi _0(t)=\left\{ \begin{array}{rl} t^3/72, &{}\quad 0\le t\le 1/4, \\ (t^3-4(t-1/4)^3)/72, &{}\quad 1/4<t\le 1/2, \\ \Psi _0(1-t), &{}\quad 1/2<t\le 1. \end{array}\right. \end{aligned}$$

For the error of $S_1$ we similarly find that

$$\begin{aligned} \frac{15}{16}\left( S_1(u,v;f)-I(u,v;f)\right) =\int _u^v \psi _1(u,v;t)f^{(4)}(t)\,{\mathrm d}t, \end{aligned}$$

where $$\psi _1(u,v;t)=h^4\Psi _1((t-u)/h)$$,

$$\begin{aligned} \Psi _1(t)=\left\{ \begin{array}{rl} 5t^3(1/3-t/2)/64, &{}\quad 0\le t\le 1/2, \\ \Psi _1(1-t), &{}\quad 1/2<t\le 1. \end{array}\right. \end{aligned}$$

Since

$$\begin{aligned} \frac{15}{16}\le \frac{\Psi _1(t)}{\Psi _0(t)}\le \frac{15}{8}, \qquad \forall t\in (0,1) \end{aligned}$$

(and both bounds are sharp), we get the desired bounds.

For the error of $S_2(u,v;f)$ we analogously find that

$$\begin{aligned} 15\,(S_2(u,v;f)-I(u,v;f))=\int _u^v \psi _2(u,v;t) f^{(4)}(t)\,{\mathrm d}t, \end{aligned}$$

where the kernel $\psi _2(u,v;t)=h^4\Psi _2((t-u)/h)$,

$$\begin{aligned} \Psi _2(t)=\left\{ \begin{array}{rl} 5t^3(1/3-t)/8, &{}\quad 0\le t\le 1/4, \\ \Psi _2(1/2-t), &{}\quad 1/4<t\le 1/2, \\ \Psi _2(t-1/2), &{}\quad 1/2<t\le 1. \end{array}\right. \end{aligned}$$

The remaining bound follows from the inequality

$$\begin{aligned} \frac{\Psi _2(t)}{\Psi _0(t)}\le 15,\qquad \forall t\in (0,1). \end{aligned}$$

**Fig. 4:**
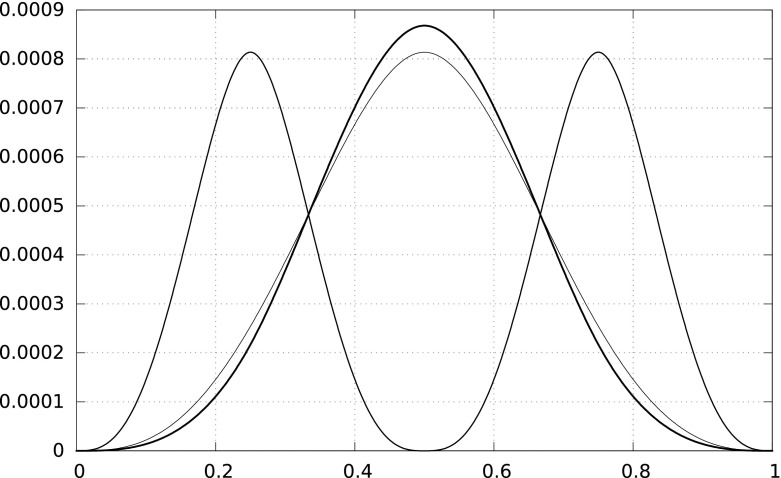
The Peano kernels $\Psi _0$ (*one hump*, *thick line*), $\Psi _1$ (*one hump*, *thin line*), and $\Psi _2$ (*two humps*). The curves intersect at $1/3$ and $2/3$

The Peano kernels $\Psi _0$, $\Psi _1$, and $\Psi _2$ are presented in Fig. 4. $\square$

In what follows we concentrate on generalizing Theorem 2 about $S_m^\mathrm{opt}$ since the other results (Theorem 1 and Proposition 1) can be treated in a similar fashion.

First we prove that the assumption $f^{(4)}>0$ in Theorem 2 can be replaced by

19 $$\begin{aligned} f^{(4)}(x)\ge 0\qquad \forall x\in [a,b]. \end{aligned}$$

### *Proof*

Suppose without loss of generality that $f^{(4)}$ is not everywhere zero in $[a,b]$. We first produce a course grid $\{z_i\}_{i=1}^{2^\ell }$ of length $(b-a)/2^\ell$ and remove from it all the points $z_i$ ($1\le i\le 2^\ell -1$) such that

$$\begin{aligned} f^{(4)}(x)=0 \qquad \forall x\in [z_{i-1},z_{i+1}]. \end{aligned}$$

Denote the successive points of the modified grid by $\{\hat{z}_i\}_{i=1}^k$, $k\le 2^\ell$. Let

$$\begin{aligned}&\displaystyle C_i=\max _{\hat{z}_{i-1}\le x\le \hat{z}_i}f^{(4)}(x),\qquad c_i=\min _{\hat{z}_{i-1}\le x\le \hat{z}_i}f^{(4)}(x),\\&\displaystyle {\mathcal J}_0=\{i:\,c_i=0\},\qquad {\mathcal J}_1=\{i:\,c_i>0\}, \quad \text{ and } \quad P_t=\bigcup _{i\in {\mathcal J}_t} [\hat{z}_{i-1},\hat{z}_i],\quad t=0,1. \end{aligned}$$

From (18) it follows that a subinterval is further subdivided if and only if $f^{(4)}\not \equiv 0$ in this subinterval. Hence for sufficiently large $m$ the coarse grid is contained in the fine grid produced by $S_m^\mathrm{opt}$ and the subintervals $[\hat{z}_{i-1},\hat{z}_i]$ with $C_i>0$ have been subdivided at least once.

Let $\hat{z}_{i-1}=x_{i,0}<\cdots <x_{i,k_i}=\hat{z}_i$ be the points of the fine grid contained in $[\hat{z}_{i-1},\hat{z}_i]$, and $h_{i,j}=x_{i,j}-x_{i,j-1}$. Define

20 $$\begin{aligned} \beta =\max _{i,j}\, S_1(x_{i,j-1},x_{i,j};f)-S_2(x_{i,j-1},x_{i,j};f). \end{aligned}$$

We now make an important observation that for any $i\in {\mathcal J_0}$ with $C_i>0$ and any $1\le j\le k_i$

21 $$\begin{aligned} \beta \le 15\,\gamma (2h_{i,j})^5C_i. \end{aligned}$$

Indeed, if this were not satisfied by a subinterval $[x_{i^*,j^*-1},x_{i^*,j^*}]$ then its predecessor, whose length is $2h_{i^*,j^*}$ and belongs to the $i^*$th subinterval of the coarse grid, would not be subdivided.

Hence, denoting by $m_0$ the number of subintervals of the fine grid in $P_0$, we have

22 $$\begin{aligned} m_0\beta \,=\,\left( m_0\beta ^{1/5}\right) ^5\,m_0^{-4}\,\le \, 15\cdot 32\,\gamma M_0^5m_0^{-4}\quad \text{ with } \quad M_0=\sum _{i\in {\mathcal J}_0}\sum _{j=1}^{k_i}h_{i,j}C_i^{1/5}.\nonumber \\ \end{aligned}$$

This implies $m_0\le 2\,(15\gamma )^{1/5}\,M_0\,\beta ^{-1/5}.$ Denoting by $m_1$ the number of subintervals of the fine grid in $P_1$, we have

23 $$\begin{aligned} m_1\beta \,=\,(m_1\beta ^{1/5})^5m_1^{-4}\,\ge \,15\,\gamma \,M_1^5m_1^{-4} \quad \text{ with } \quad M_1=\sum _{i\in {\mathcal J}_1}\sum _{j=1}^{k_i}h_{i,j}c_i^{1/5},\qquad \end{aligned}$$

which implies $m_1\,\ge \,(15\gamma )^{1/5}\,M_1\,\beta ^{1/5}.$ Hence $m_0/m_1\le 2M_0/M_1$ and this bound is independent of $m$. However it depends on $\ell$. Taking $\ell$ large enough we can make $m_0/m_1$ arbitrarily small.

From Lemma 1 it follows that the integration error in $P_0$ is upper bounded by $m_0\beta$. Since $f^{(4)}$ is positive in $P_1$, the error in $P_1$ is asymptotically (as $m\rightarrow \infty$) lower bounded by $m_1\beta /(15\cdot 32)$. Hence for $\ell$ large enough the error in $P_0$ is arbitrarily small compared to that in $P_1$. In addition, the error in $P_1$ follows the upper bound of Theorem 2. The proof is complete. $\square$

We now pass to functions with endpoint singularities. To fix the setting, we assume that $f$ is continuous in the closed interval $[a,b]$ and $f\in C^4([a_1,b])$ for all $a<a_1<b$. Moreover,

$$\begin{aligned} \lim _{x\rightarrow a} f^{(4)}(x)=+\infty , \end{aligned}$$

and this divergence is asymptotically monotonic, i.e., there is $\delta >0$ such that

$$\begin{aligned} f^{(4)}(x_1)\ge f^{(4)}(x_2)>0 \quad \text{ for } \text{ all } \;a<x_1\le x_2\le a+\delta . \end{aligned}$$

As before, we prove that for such functions the upper error bound for $S_m^\mathrm{opt}$ in Theorem 2 is still valid.

### *Proof*

First, we observe that the difference $S_1(a,a+h;f)-S_2(a,a+h;f)$ converges to zero faster than $h$. Indeed, in view of (18) we have

24 $$\begin{aligned} \nonumber S_1(a,a+h;f)-S_2(a,a+h;f)&= h\,\int _a^{a+h}h^3\Psi _0((t-a)/h)f^{(4)}(t)\,{\mathrm d}t \\&\le h\,\int _a^{a+h}\Psi _0(t-a)f^{(4)}(t)\,{\mathrm d}t. \end{aligned}$$

This assures that the partition is denser and denser in the whole $[a,b]$ and the integration error goes to zero.

Second, we have that $L^\mathrm{opt}(f)<\infty$. Indeed, by Hölder’s inequality

$$\begin{aligned} \int _a^b (f^{(4)}(x))^{1/5}\,{\mathrm d}x&= \int _a^b (x-a)^{-3/5}\left( (x-a)^3f^{(4)}(x)\right) ^{1/5} \\&\le \left( \int _a^b (x-a)^{-3/4}\,{\mathrm d}x\right) ^{4/5} \left( \int _a^b (x-a)^3f^{(4)}(x)\,{\mathrm d}x\right) ^{1/5}, \end{aligned}$$

which is finite due to (16).

Now, let $\ell$ be such that $(b-a)2^{-\ell }\le \delta$, and let $\{z_i\}_{i=-\infty }^k$ with $k=2^\ell -1$ be the (infinite) coarse grid defined as

$$\begin{aligned} z_i=\left\{ \begin{array}{rl} a+(b-a)2^{-\ell +i}, &{}\quad i\le -1, \\ a+(b-a)2^{-\ell }(i+1), &{}\quad 0\le i\le k. \end{array}\right. \end{aligned}$$

Denote, as before, $C_i=\max _{z_{i-1}\le x\le z_i}f^{(4)}(x)$. We obviously have $C_i=f^{(4)}(z_{i-1})>0$ for all $i\le 0$. For simplicity, we also assume $C_i>0$ for $1\le i\le k$.

Let $m$ be sufficiently large so that the fine grid produced by $S_m^\mathrm{opt}$ contains all the points $z_i$ for $i\ge 0$. Moreover, we can assume that each subinterval $[z_{i-1},z_i]$ with $i\ge 1$ has been subdivided at least once. Let $[a,z_{-s}]$ be the first subinterval of the fine grid.

Let us further denote $P_0=[a,z_0]$ and $P_1=[z_0,b]$. Then $P_0=P_{0,0}\cup P_{0,1}$ where $P_{0,0}$ consists of $[0,z_{-s}]$ and all subintervals of the course grid that have not been subdivided by $S_m^\mathrm{opt}$. Let $m_{0,0}$, $m_{0,1}$, $m_1$ be the numbers of subintervals of the fine grid in $P_{0,0}$, $P_{0,1}$, $P_1$, respectively.

Define $\beta$ as in (20). In view of (24), the distance $(z_{-s}-a)$ decreases slower than $\beta$ as $m\rightarrow \infty$, and therefore $m_{0,0}$ is at most proportional to $\log _2(1/\beta )$. Since (21) holds for the subintervals in $P_{0,1}$, the number $m_{0,1}$ can be estimated as $m_0$ in (22) with

$$\begin{aligned} M_0\,\le \,\sum _{i=-s+1}^0(z_i-z_{i-1})C_i^{1/5} \,\le \, 2\int _0^{z_{-1}}\left( f^{(4)}(x)\right) ^{1/5}\,{\mathrm d}x, \end{aligned}$$

where the last inequality follows from monotonicity of $f^{(4)}$. Since $m_1$ can be estimated as in (23) we obtain, analogously to the previous proof, that the number of subintervals in $P_1$ and the error in $P_1$ dominate the scene. The proof is complete. $\square$

We stress that for continuous functions with endpoint singularities we always have $L^\mathrm{opt}(f)<\infty$, which is not true for $L^\mathrm{std}(f)$. An example is provided by

$$\begin{aligned} f(x)=\int _x^1\frac{(t-x)^3}{3!}f^{(4)}(t)\,{\mathrm d}t,\qquad 0\le t\le 1, \end{aligned}$$

with $f^{(4)}(x)=(t\ln t)^{-4}.$ Indeed, since $f(0)=\int _0^1(3!\,t\ln ^4t)^{-1}\,{\mathrm d}t<\infty$, the function is well defined and $L^\mathrm{opt}(f)<\infty$, but

$$\begin{aligned} L^\mathrm{std}(f)=\int _0^1\frac{-1}{t\ln t}\,{\mathrm d}t=\infty . \end{aligned}$$

For such functions, the subdivision process of $S_m^\mathrm{std}$ will not collapse [which follows from (24)] and the error will converge to zero; however, the convergence rate $m^{-4}$ will be lost. On the other hand, if $L^\mathrm{std}(f)<\infty$ then the error bounds of Theorem 1 hold true.

### *Example 3*

Consider the integral

$$\begin{aligned} \int _0^1 (p+1)\,x^p\,{\mathrm d}x. \end{aligned}$$

**Fig. 5:**
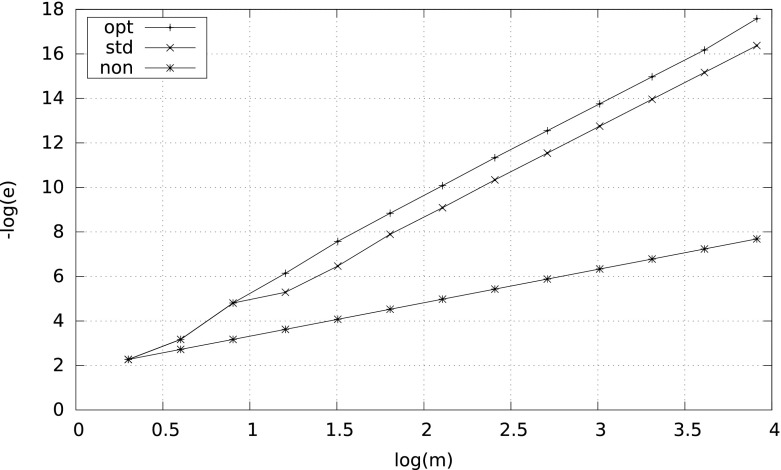
Error $e$ versus $m$ for $\int _0^1\tfrac{3}{2}\sqrt{x}\,{\mathrm d}x$

**Fig. 6:**
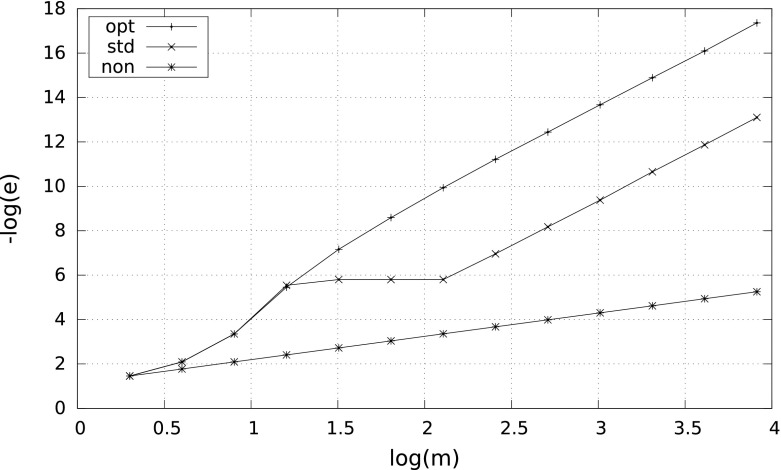
Error $e$ versus $m$ for $\int _0^1\tfrac{21}{20}\root 20 \of {x}\,{\mathrm d}x$

The integrand is continuous at $0$ only if $p\ge 0$. Then both, $L^\mathrm{opt}(f)$ and $L^\mathrm{std}(f)$, are finite. However, $L^\mathrm{non}(f)<\infty$ only if $p$ is a non-negative integer or $p>3$. Figures 5 and 6, where the results for $p=1/2$ and $p=1/20$ are presented, show that, indeed, the adaptive quadratures $S_m^\mathrm{std}$ and $S_m^\mathrm{opt}$ converge as $m^{-4}$, and $S_m^\mathrm{non}$ converges at a very poor rate.

**Fig. 7:**
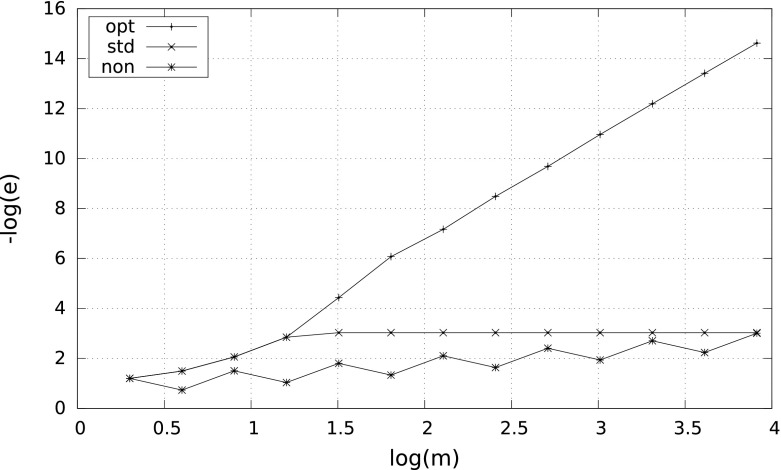
Error $e$ versus $m$ for function $f$ of Example 4

We end this paper by showing the importance of continuity of $f$.

### *Example 4*

Consider the integral $\int _a^b f(x)\,{\mathrm d}x$ with $a=-1/2$, $b=1$,

$$\begin{aligned} f(x)=\left\{ \begin{array}{rl} 0, &{}\quad -1/2\le x\le 0, \\ x^{-1/2}/2, &{}\quad 0<x\le 1. \end{array}\right. \end{aligned}$$

In this case $L^\mathrm{opt}(f)<\infty$ but $L^\mathrm{std}(f)=\infty$. Figure 7 shows that $S_m^\mathrm{opt}$ enjoys the ‘right’ convergence $m^{-4}$ but $S_m^\mathrm{std}$ completely fails. This is because the critical value

$$\begin{aligned} \max _i\; S_1\left( x_{i-1}^{(m)},x_i^{(m)};f\right) -S_2\left( x_{i-1}^{(m)},x_i^{(m)};f\right) \end{aligned}$$

does not converge faster than $h$; the algorithm keeps dividing the subinterval containing $0$. As a result, the standard adaptive algorithm is asymptotically even worse than the nonadaptive algorithm.

Equally striking is the difference between OPTIMAL and STD. While OPTIMAL works perfectly well, see Table 6, STD will never reach the stopping criterion for $\varepsilon \le 10^{-3}$, and will loop forever.

Unfortunately, this example is misleading. The very good behavior of $S_m^\mathrm{opt}$ is a consequence of our “lack of bad luck” rather than a rule. Indeed, it is enough to change the value of $f$ in $[-1/2,0]$ from $0$ to $7/3$ to see that then $S_1(a,(a+b)/2;f)-S_2(a,(a+b)/2;f)=0$ although $\int _a^{(a+b)/2}f(x)\,{\mathrm d}x=19/12>0$. As a consequence, $\lim _{m\rightarrow \infty }S_m^\mathrm{opt}(f)=13/6$ while the integral equals $25/12$.

**Table 6:** Optimal quadrature for function $f$ of Example 4

$\varepsilon$	$\text{ Optimal }\;(\mathrm{B}=4\sqrt{2})$	
	Error	$m$
1.0E$-$03	$-$9.48044E$-$04	35
1.0E$-$04	$-$3.68769E$-$05	63
1.0E$-$05	6.29168E$-$06	107
1.0E$-$06	5.83337E$-$07	175
1.0E$-$07	6.16070E$-$08	281
1.0E$-$08	6.16381E$-$09	467
1.0E$-$09	5.46528E$-$10	797
1.0E$-$10	5.50559E$-$11	1,377
1.0E$-$11	5.89940E$-$12	2,375
1.0E$-$12	6.17718E$-$13	4,131
